# In lupus cystitis, is the urinary tract dilated or obstructed?

**DOI:** 10.1002/iid3.777

**Published:** 2023-02-09

**Authors:** Yoshiyuki Abe, Nozomi Kawamata, Ayako Makiyama, Takeshi Ashizawa, Takuo Hayashi, Naoto Tamura

**Affiliations:** ^1^ Department of Internal Medicine and Rheumatology Juntendo University School of Medicinen Tokyo Japan; ^2^ Department of Urology Juntendo University School of Medicine Tokyo Japan; ^3^ Department of Human Pathology Juntendo University School of Medicine Tokyo Japan

**Keywords:** animals, diseases, human, systemic lupus erythematosus

## Abstract

**Objectives:**

Lupus cystitis is a rare but serious complication of systemic lupus erythematosus (SLE) that can cause permanent bladder dysfunction, leading to irreversible deterioration of kidney function. We report two cases of SLE with lupus cystitis who showed different images from the same cause of disease.

**Methods:**

Patient 1, a 67‐year‐old woman diagnosed with SLE presented with persistent dysuria for 3 weeks with sudden headache and vomiting. She was hospitalized because of acute kidney injury; the serum creatinine level was 10.68 mg/dL. Computed tomography (CT) showed significant bilateral ureteral stenosis and bilateral hydronephrosis. Patient 2, a 45‐year‐old woman diagnosed with SLE presented with dysuria requiring self‐catheterization. CT showed significant bilateral ureteral dilatation and bilateral hydronephrosis.

**Results:**

In patient 1, the right kidney was afunctional. Left nephrostomy was performed on Day 2. Her serum creatinine returned to the normal range. In patient 2, After admission, she changed to an indwelling bladder catheter. Her serum creatinine level improved from 2.04 to 1.31 mg/dL.

**Conclusion:**

In patients with lupus cystitis, the urinary tract is commonly dilated, but stenosis has been seen in rare case. Physicians should be careful in diagnosing it.

## Graphical abstract

Dilated or obstructed urinary tract in two cases with lupus cystitis.

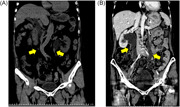

## 1 INTRODUCTION

Lupus cystitis is a rare but serious complication of systemic lupus erythematosus (SLE) that can cause permanent bladder dysfunction, leading to irreversible deterioration of kidney function.^1^ We report two cases of SLE with lupus cystitis who showed different images from the same cause of disease.

## 2 CASE PRESENTATION

**Figure 1:**
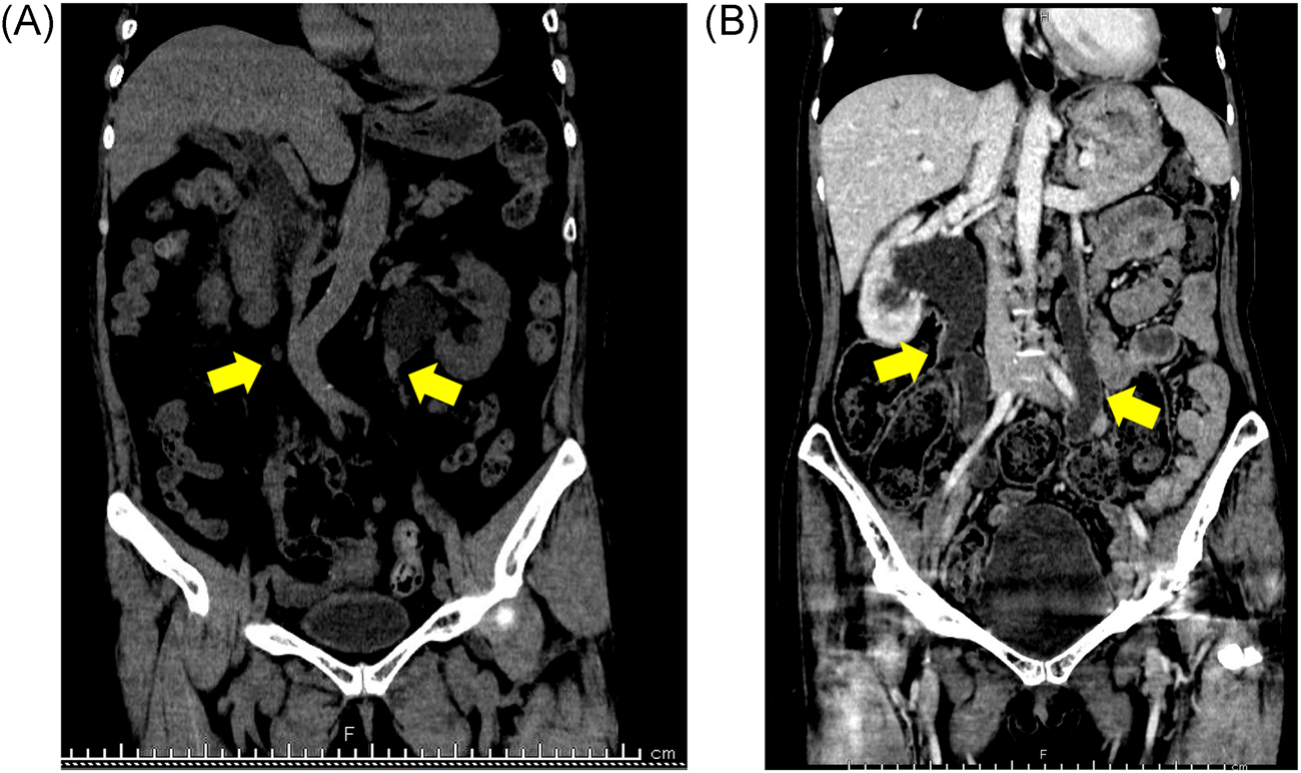
(A) Significant bilateral ureteral stenosis (yellow arrows) and bilateral hydronephrosis. (B) Significant bilateral ureteral dilatation and bilateral hydronephrosis.

These two cases were patients with SLE, and they hospitalized because of acute kidney injury due to bilateral hydronephrosis. They showed different images from the same cause of disease. Patient 1, a 67‐year‐old woman diagnosed with SLE (polyarthritis dominant) overlapped with limited‐type systemic sclerosis at age 15 years, presented with persistent dysuria for 3 weeks with sudden headache and vomiting. She was hospitalized because of acute kidney injury; the serum creatinine level was 10.68 mg/dL. Computed tomography (CT) showed significant bilateral ureteral stenosis and bilateral hydronephrosis (Figure 1A). Patient 2, a 45‐year‐old woman diagnosed with SLE at age 28 years (serositis dominant), presented with dysuria requiring self‐catheterization. Initially, she prioritized fertility treatment and refused immunosuppressive therapy. However, 4 years later, immunosuppressive therapy was started for ileus due to lupus enteritis and bilateral hydronephrosis due to lupus cystitis. Uroflowmetry at the age of 40 revealed the urine volume of 149.3 mL, the maximum urine flow rate of 5.9 mL/s, and the residual urine volume of 109 mL, suggesting urination disorder due to lupus cystitis. At the age of 46, the postvoid residual urine volume was 343 mL. She refused cystoscopy and bladder biopsy. CT showed significant bilateral ureteral dilatation and bilateral hydronephrosis (Figure 1B).

**Figure 2:**
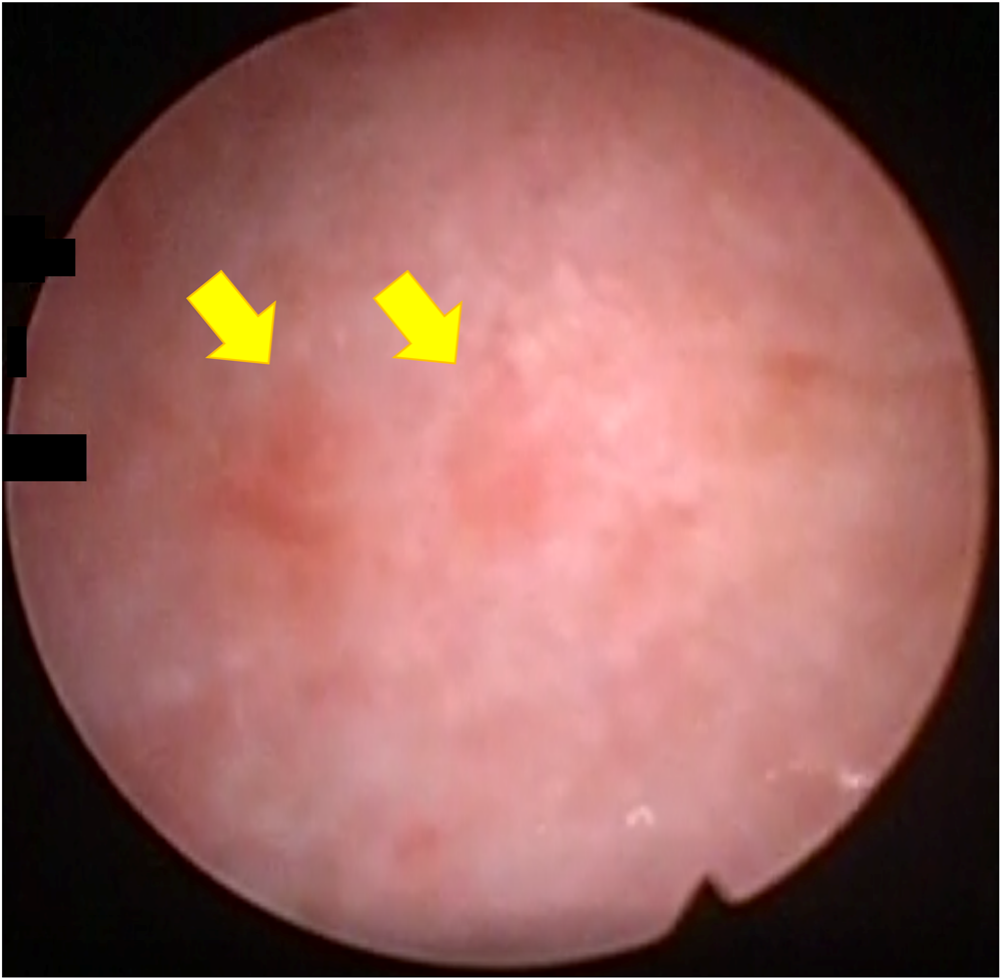
Cystoscopic findings. Patient 1 shows the multiple reddish bladder mucosal lesions (yellow arrows).

**Figure 3:**
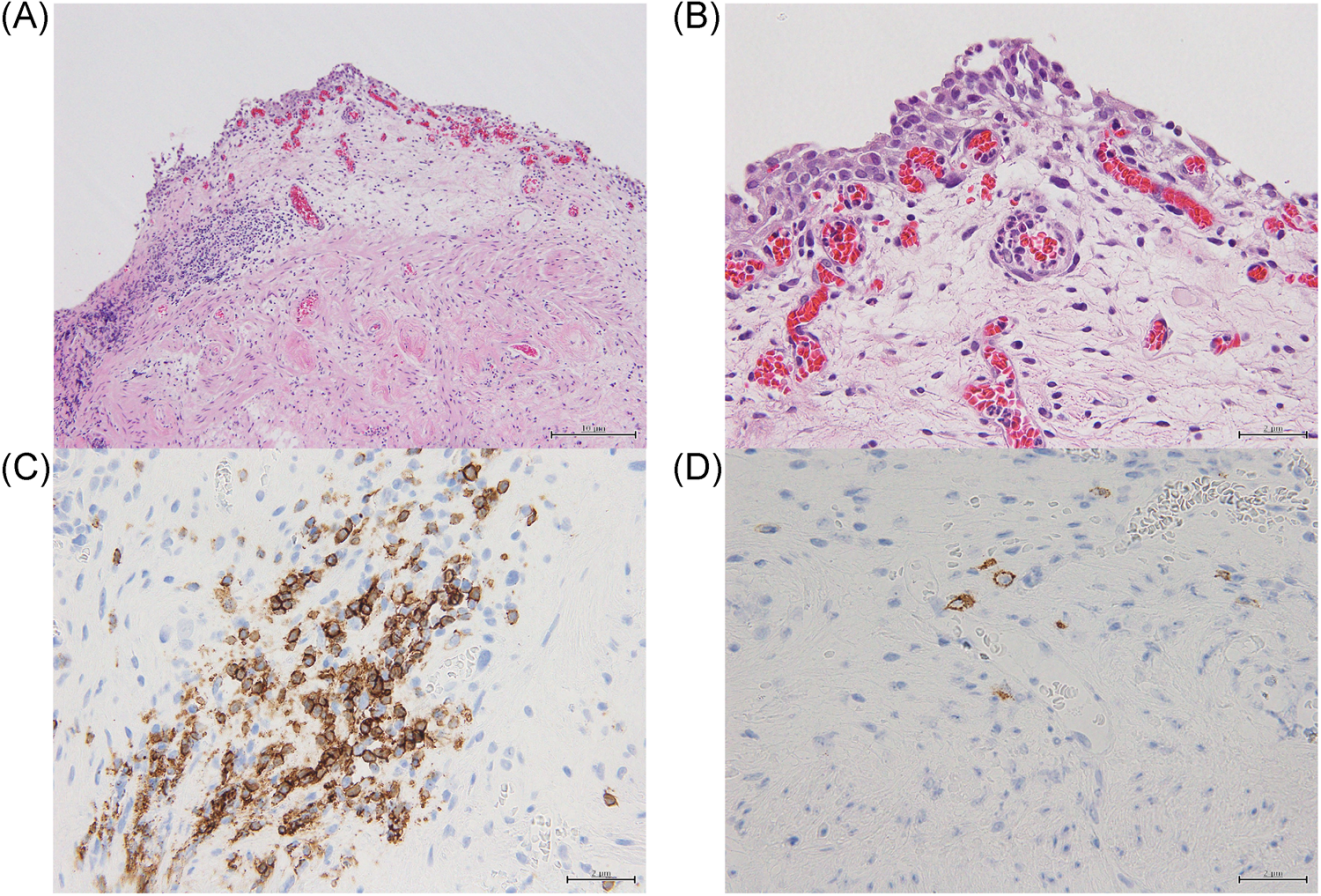
Histopathological images of bladder biopsy in patient 1. (A, B) Dense lymphoplasmacytic cell infiltration with increased plasma cell, epithelial denudation, and stromal edema and fibrosis on hematoxylin & eosin (H&E) staining (A, low power field; B, high power field). (C) Immunohistochemistry of CD 20 was performed. B lymphocyte infiltration were shown. (D) Immunohistochemistry of CD 138 was performed. Plasma cell infiltration were shown.

In patient 1, the right kidney was afunctional. Left nephrostomy was performed on Day 2. Her serum creatinine returned to the normal range. We performed the retrograde pyelography on Day 19. However, neither the catheter nor even the guidewire had passed into both ureters, and we concluded that ureteral stenoses were from the ureteropelvic junction to ureterovesical junction on both. Figure 2 shows cystoscopic findings that the multiple reddish bladder mucosal lesions. She was discharged on Day 38. Bladder biopsy demonstrated interstitial cystitis associated with SLE such as dense lymphocytic cell infiltration with increased plasma cells, epithelial denudation, and stromal edema and fibrosis (Figure 3). In patient 2, After admission, she changed to an indwelling bladder catheter. Her serum creatinine level improved from 2.04 to 1.31 mg/dL. She was discharged on Day 13.

## 3 DISCUSSION

Chronic and unresolved lupus cystitis can cause structural changes in the urinary tract because dysfunction of the ureterovesical junction caused reflux.^2^ In patient 2, we considered that bilateral ureteral dilation and hydronephrosis resulted from just the bladder contraction. In patients with lupus cystitis, the urinary tract is commonly dilated^3^, ^4^, ^5^ but stenosis has been seen in rare case.^6^, ^7^ Physicians should be careful in diagnosing it.

## AUTHOR CONTRIBUTIONS

**Yoshiyuki Abe**: Conceptualization (lead); writing – original draft (lead); formal analysis (lead); writing – review and editing (equal). **Nozomi Kawamata**: Collected data, review and editing (equal). **Ayako Makiyama**: Collected data, review and editing (equal). **Takeshi Ashizawa**: Collected data, review and editing (equal). **Takuo Hayashi**: Collected data, review and editing (equal). **Naoto Tamura**: Conceptualization, review and editing (equal).

## CONFLICT OF INTEREST STATEMENT

The authors declare no conflict of interest.

## Data Availability

The data that support the findings of this study are available from the corresponding author upon reasonable request.

## References

[1] Weisman MH , McDanald EC , Wilson CB . Studies of the pathogenesis of interstitial cystitis, obstructive uropathy, and intestinal malabsorption in a patient with systemic lupus erythematosus. Am J Med. 1981;70(4):875‐881. 7211922 10.1016/0002-9343(81)90547-7

[2] Koh JH , Lee J , Jung SM , et al. Lupus cystitis in Korean patients with systemic lupus erythematosus: risk factors and clinical outcomes. Lupus. 2015;24(12):1300‐1307. 26038343 10.1177/0961203315588575

[3] Hong S , Kim YG , Ahn SM , et al. Clinical outcomes of hydronephrosis in patients with systemic lupus erythematosus. Int J Rheum Dis. 2016;19(12):1317‐1321. 26224134 10.1111/1756-185X.12599

[4] Santacruz JC , Pulido S , Arzuaga A , Mantilla MJ , Londono J . Lupus cystitis, from myth to reality: a narrative review. Cureus. 2021;13(12):e20409. 35047251 10.7759/cureus.20409 PMC8757392

[5] John K , Varughese K , Boaz RJ , George T . Lupus cystitis: unusual cause of renal failure in systemic lupus erythematosus. BMJ Case Rep. 2019;12(12):e233446. 10.1136/bcr-2019-233446 PMC6936437 31862817

[6] Kaneshita S , Kishimoto M , Okada M . Lupus enteritis and cystitis. Intern Med. 2017;56(4):467‐468. 28202875 10.2169/internalmedicine.56.7781 PMC5364206

[7] Takezawa Y , Ohtake N , Nakano K , Yamanaka H . Lupus cystitis. Report of a case. Hinyokika Kiyo. 1994;40(8):717‐720. 7942371

